# Next-Generation Molecular Investigations in Lysosomal Diseases: Clinical Integration of a Comprehensive Targeted Panel

**DOI:** 10.3390/diagnostics11020294

**Published:** 2021-02-12

**Authors:** Bénédicte Sudrié-Arnaud, Sarah Snanoudj, Ivana Dabaj, Hélène Dranguet, Lenaig Abily-Donval, Axel Lebas, Myriam Vezain, Bénédicte Héron, Isabelle Marie, Marc Duval-Arnould, Stéphane Marret, Abdellah Tebani, Soumeya Bekri

**Affiliations:** 1 Department of Metabolic Biochemistry, Rouen University Hospital, 76000 Rouen, France; b.sudrie-Arnaud@chu-rouen.fr (B.S.-A.); sarah.snanoudj@chu-rouen.fr (S.S.); helene.dranguet@chu-rouen.fr (H.D.); abdellah.tebani@chu-rouen.fr (A.T.); 2 Normandie Univ, UNIROUEN, CHU Rouen, INSERM U1245, 76000 Rouen, France; ivana.dabaj@chu-rouen.fr (I.D.); lenaig.donval@gmail.com (L.A.-D.); stephane.marret@chu-rouen.fr (S.M.); 3 Department of Neonatal Pediatrics, Intensive Care and Neuropediatrics, Rouen University Hospital, 76000 Rouen, France; 4 Department of Neurophysiology, Rouen University Hospital, 76031 Rouen, France; axel.lebas@chu-rouen.fr; 5 Normandie Univ, UNIROUEN, INSERM U1245, Department of Genetics and Reference Center for Developmental Disorders, Rouen University Hospital, Normandy Center for Genomic and Personalized Medicine, 76000 Rouen, France; myriam.vezain@inserm.fr; 6 Centre de Référence des Maladies Lysosomales, Service de Neurologie Pédiatrique, CHU Armand Trousseau-La Roche Guyon, GHUEP, APHP, 75000 Paris, France; benedicte.heron@aphp.fr; 7 Center for Lysosomal Diseases, Pediatric Neurology Department, UH Armand Trousseau-La Roche Guyon, APHP, GUEP, 75000 Paris, France; 8 Department of Internal Medicine, Rouen University Hospital, 76000 Rouen, France; isabelle.marie@chu-rouen.fr; 9 Department of Pediatrics, Bicetre Hospital, APHP, 75000 Paris, France; marc.duval-arnould@aphp.fr

**Keywords:** NGS, next generation sequencing, inborn errors of metabolism, lysosomal disorders

## Abstract

Diagnosis of lysosomal disorders (LDs) may be hampered by their clinical heterogeneity, phenotypic overlap, and variable age at onset. Conventional biological diagnostic procedures are based on a series of sequential investigations and require multiple sampling. Early diagnosis may allow for timely treatment and prevent clinical complications. In order to improve LDs diagnosis, we developed a capture-based next generation sequencing (NGS) panel allowing the detection of single nucleotide variants (SNVs), small insertions and deletions, and copy number variants (CNVs) in 51 genes related to LDs. The design of the LD panel covered at least coding regions, promoter region, and flanking intronic sequences for 51 genes. The validation of this panel consisted in testing 21 well-characterized samples and evaluating analytical and diagnostic performance metrics. Bioinformatics pipelines have been validated for SNVs, indels and CNVs. The clinical output of this panel was tested in five novel cases. This capture-based NGS panel provides an average coverage depth of 474× which allows the detection of SNVs and CNVs in one comprehensive assay. All the targeted regions were covered above the minimum required depth of 30×. To illustrate the clinical utility, five novel cases have been sequenced using this panel and the identified variants have been confirmed using Sanger sequencing or quantitative multiplex PCR of short fluorescent fragments (QMPSF). The application of NGS as first-line approach to analyze suspected LD cases may speed up the identification of alterations in LD-associated genes. NGS approaches combined with bioinformatics analyses, are a useful and cost-effective tool for identifying the causative variations in LDs.

## 1. Introduction

The lysosome is an intracellular organelle characterized by its acidic pH, and its main function consists in degradation of intra or extracellular macromolecules into monomers. This metabolic process is carried out by more than fifty lysosomal enzymes. Additionally, over a hundred structural proteins and carriers essential for lysosomal function have been identified [1]. “Lysosomal storage disorders” (LSD) was the conventional term used to describe the group of inborn errors of metabolism (IEMs) related to the absence or failure of substrate degradation or transport, and their subsequent accumulation in the lysosome [2]. However, in recent years, the lysosome is being viewed as a dynamic structure with multiple roles in nutrient sensing, autophagy, apoptosis, and cellular response to environmental cues. It is also a signaling hub that interacts with other organelles [3]. In this context, the chosen term has shifted to lysosomal disorders (LDs) instead of LSD to better reflect the complexity of these diseases. In LDs, the inheritance pattern is autosomal recessive except for three disorders (Fabry, Danon, and Hunter diseases) which are X-linked. Clinical presentations of LDs vary greatly, and age at onset ranges from the antenatal period all the way to adulthood. However, in some cases, cardinal signs may steer clinical physicians towards a particular disorder, such as specific dysmorphic features, ocular or articular involvement, organomegaly, multiple dysostosis, valvulopathy, neurological defects or psychomotor delay. An early diagnosis allows an appropriate medical care, as many specific treatments have recently been developed, and thus reduces morbidity [4,5]. Currently, biological diagnosis relies on a three-phase process: (i) characterization of accumulated metabolites, (ii) enzyme activity assessment, and (iii) molecular investigations. Additionally, in some cases, molecular study as first-line exploration is mandatory to reach the diagnosis. For instance, in case of X-linked pathologies such as Fabry disease, the measurement of enzyme activity may fail to identify heterozygous females due to X inactivation process. Besides, in some autosomal disorders, such as most of neuronal ceroid lipofuscinosis (NCL), no biological tests are available and molecular approaches are the only diagnostic option. 

The rise of “omics-based” approaches and the tremendous technological shift, in both multiscale biological information capture and data management, offer a remarkable opportunity to change the ways we screen, diagnose, treat, and monitor inherited metabolic diseases [5,6,7]. Next generation sequencing (NGS) technologies represent an essential tool for rapid and effective diagnosis of these diseases and may be used in some complex situations prior to multiple and often sequential functional studies. Recent studies highlighted the clinical utility of NGS approach for LD genetic diagnosis [8,9,10,11]. Here we report on the design, validation and testing of an NGS panel for genes involved in LDs named LysoGene.

## 2. Materials and Methods

### 2.1. Patients

Twenty-one well-characterized LD patients have been included for validation purposes (Supplementary Table S1). Twenty-seven disease-causing variations and 50 benign variations have been previously identified by Sanger sequencing and were used for validation of the single nucleotide variants (SNVs) and small insertions/deletions (indels) sequencing process and the bioinformatics pipeline (Supplementary Tables S1 and S2). To illustrate the clinical utility of this panel, five LD patients are reported. 

Case 1: A female child presented at 3 months of age with severe organomegaly (hepatomegaly at 6 cm and splenomegaly at 9 cm), associated with severe malnutrition, without diarrhea. No dysmorphy was noted. The liver biopsy was in favor of a storage disease.

Case 2: This female child was born at term from a non-consanguineous couple, eutrophic after a normal pregnancy, and with a good adaptation to extra-uterine life. At the age of two and a half years old, she presented with a speech delay and a flat tympanogram and transtympanic ventilation tube was inserted. At 3 years old, she was hospitalized for seizures with predominantly right occipital spikes on the electroencephalogram (EEG) wake and sleep patterns. A second episode of seizures induced by hyperthermia occurred a few months later. She had a disturbed sleep pattern with repeated awakenings, agitation and crying, sensory dysregulation including severe agitation and intolerance to loud noises, and poor communication. Brain MRI showed a retrocerebellar arachnoid cyst and cerebellar atrophy. Based on these elements, late infantile neuronal ceroid lipofuscinosis (CLN2, CLN5, CLN6 or CLN7) was suspected.

Case 3: This was the third child of a couple, born prematurely at 35 weeks of gestation by caesarean section for abnormal fetal heart rhythm. She was hospitalized at 3 months of age for psychomotor regression with decrease of focus and ocular following of objects and persons, as well as axial hypotonia. High blood pressure was diagnosed in the emergency department, and the child was put on calcium channel blocker. The MRI and the EEG showed no anomalies. A cherry red macula was found on ophthalmological examination. A LysoGene panel was requested.

Case 4: The patient was the second child of healthy non-consanguineous parents. Pregnancy was without particularity with a birth weight of 2830 g, a birth length of 47 cm and a head circumference of 34 cm. He was hospitalized in the neonatal intensive care for amniotic fluid aspiration associated with patent ductus arteriosus and suspicion of neonatal infection. This child acquired walking at around 12 months old, day and night cleanliness at 4 years old. At two and a half years old, he was treated for bilateral serous otitis media revealed by a hoarse voice and difficulties understanding. At three years old, he did not pronounce words properly and only formed simple sentences. He had a behavioral disorder with aggressiveness, concentration difficulties and disabling headaches. At 5 years old, he had a height and weight at + 1SD and presented with signs of storage such as square face, skin thickening, and enlarged joints and bone. At the metabolic level, elevated urinary excretion of heparan sulfate and a decreased activity in Heparan-alpha-glucosaminide *N*-acetyltransferase were consistent with Sanfilippo type C (Mucopolysaccharidosis type IIIC) diagnosis. The *HGSNAT* gene was analyzed using Sanger sequencing and two pathogenic variants were identified in the heterozygous state: a splicing variant (NM_152419.2:c.234+1G>A-p.?) resulting in a modification of the exon 2 splicing, and a missense variant NM_152419.2:c.710C>A-p.(Pro237Gln). Both variants are reported in the Human Gene Mutation Database (HGMD) and have been published [12]. However, allelic segregation analysis showed that both variants were inherited from the mother who was clinically healthy. Of note, the DNA sample from the father was not available to us. We decided to investigate this case using the LysoGene panel to unveil the alteration inherited from the father. 

Case 5: A 31-year-old patient presented with diffuse myalgia. He had progressive exercise intolerance during the last 5 years. He also suffered from sleep apnea. The patient had been hospitalized several times and underwent many explorations without any diagnosis having been reached. Classical neuromuscular work up was normal, including electromyogram (EMG) and creatine phosphokinase (CPK). 

Written informed consents were obtained from the parents when the patient is under 18 or from the adult patient in order to perform any investigation related to their pathology.

### 2.2. NGS Sequencing 

DNA extraction: for NGS analysis, blood genomic DNAs were extracted using a silica-membrane-based DNA purification method (QIAamp DNA Blood Mini Kit, QIAGEN). NGS sequencing was performed in the IRIB-Rouen University Hospital Facility (Service Commun de Génomique).

Gene panel design: our approach aimed to capture, and sequence 51 genes implicated in LD (Table 1, Supplementary Table S3). Five additional genes were included for identity monitoring of patients (*CCDC88C*, *NIPBL*, *MLH1*, *APC*, *PTEN*). The design of the LysoGene panel covered the coding regions, the promoter region and the flanking intronic sequences for 43 genes. In addition, 3′ untranslated sequences were included for 2 genes (*AGA* and *ARSA*), and the entire gene sequences were covered for 6 genes (*ARSB*, *CLN3*, *CLN8*, *IDS*, *SGSH*, and *NAGLU*). In total, 708 regions were targeted including 506 exonic regions. Custom primers were designed using the SureDesign software (Agilent Technologies, Santa Clara, CA, USA).

Library preparation and sequencing: the library preparation protocol was set up using the QXT SureSelect enrichment kit from Agilent. Library construction was done using enzymatic fragmentation and the SureSelectQXT kit (Agilent Technologies, Santa Clara, CA, USA) to capture targeted sequences. Patients’ libraries were pooled after the enrichment step. The protocol was either performed manually or automated on a Sciclone NGSx workstation (PerkinElmer, Waltham, MA, USA). Libraries were sequenced on a MiSeq or a NextSeq 500 platform (Illumina, San Diego, CA, USA) using 2 × 150 bp paired-end sequencing. 

Bioinformatics pipelines: for the detection of SNVs, indels and copy number variants (CNVs), a double bioinformatics pipeline was used with complementary algorithms in order to optimize the disease-causing variant detection rate:

(i) The bcl2fastq conversion software (Illumina, v2.20) was used for reads demultiplexing and generation of Fastq files. Sequenced reads were mapped to the human reference sequence (GRCh37, Hg19) using the Burrows–Wheeler Aligner (BWA v.0.7.17). Read duplicates were marked with Picard tools (v2.18.0), local realignments around indels, base-quality-score recalibration and variant calling were performed with the Genome Analysis Toolkit (GATK 4.0.6.0). Single-nucleotide variants and small indels were identified with the GATK HaplotypeCaller (v4.0.6.0), VarScan2 (v2.4.3) and Vardict (v1.5.1). Variants were then annotated with SnpEff (v.4.2) and Alamut-batch (v.1.12).
(ii) The second pipeline, large-scale rearrangements and the related CNVs were detected using the CANOES and GRIDSS software [13,14,15].

For each sequencing run, PDF quality reports integrating the number of clusters/mm^2^, percentage of bases with a Qscore > 30, FastQC reports, percentage of mapped, reads, on- and off-targets percentages, percentage of covered bases and mean sequencing depth were automatically generated using the in-house tool PyQua (Python Qualitics). 

Data analysis: An in-house software, CanDiD allowed for the prioritization and filtration of variants using defined criteria such as minor allele frequency in public databases or consequences of the variant (missense, synonym, nonsense, splicing). The filtered variants were compared to variant databases including dbSNP (https://www.ncbi.nlm.nih.gov/snp/ (accessed on 10 January 2021)), GnomAD (https://gnomad.broadinstitute.org/ (accessed on 10 January 2021)), HGMD (http://www.hgmd.cf.ac.uk/ (accessed on 10 January 2021)), LOVD (https://databases.lovd.nl/shared/genes (accessed on 10 January 2021)), and gene specific databases such as NPC-db2 (https://medgen.medizin.uni-tuebingen.de/NPC-db2/ (accessed on 10 January 2021)), Pompe variant database (http://www.pompevariantdatabase.nl/ (accessed on 10 January 2021)), and dbFGP (http://www.dbfgp.org/dbFgp/fabry/Mutation.html (accessed on 10 January 2021)).

**Figure 1:**
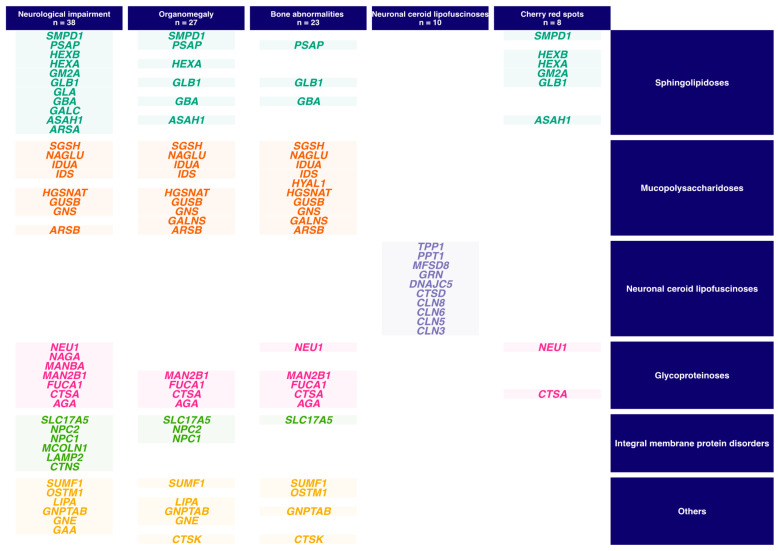
Overview of the genes included in the different LysoGene sub-panels.

The analysis of the captured sequence takes into account the clinical context. In this perspective, we defined five overlapping sub-panels for sequence analysis (Figure 1): Organomegaly (27 genes), neurological impairment (38 genes), bone abnormalities (23 genes), neuronal ceroid lipofuscinoses (10 genes), and cherry red spots (8).

Evaluation of the pathogenicity of the variants were analyzed with in silico tools such as SIFT [16], PolyPhen2 [17] or MutationTaster [18] and M-CAP [19] to predict potential deleterious effect on protein function, and HumanSplicingFinder 2.4.1 [20], MaxEntScan [21], NNSPLICE [22], GeneSplicer [23], SpliceSiteFinder [24], and ESEFinder [25] for possible effect on splicing. Variant classification was done according to the recommendations of the American College of Medical Genetics [26].

The control of the sample identity was performed using a multiplex SNaPshot analysis comparing five SNPs located within the captured regions of 5 genes unrelated to LDs included in the panel. To validate the panel in a diagnostic context, analytical accuracy, intra-assay and inter-assay reproducibility were assessed. 

## 3. Results

### 3.1. Quality Metrics

The NGS assay provided an average read depth of 474×. This deep coverage allowed for simultaneous detection of SNVs and CNVs in one comprehensive analysis. All the targeted regions were covered above the minimum depth required of 30×.

### 3.2. Panel Performances for the Detection of SNVs and Indels

Accuracy: The concordance between this panel results and the reference data was 100% for all 77 variants. Thus, the detection of these variants has been achieved with 100% analytic sensitivity. 

Intra- and inter-assay reproducibility: the ratios between the values obtained for all metrics measured in the samples used for intra- and interassay reproducibility tests were equal or close to 1 (Supplementary Tables S4 and S5) demonstrating the consistency of the results. 

### 3.3. Panel Performances for the Detection of CNVs 

For CNVs, the performances of the in-house bioinformatics tool, CANOES, for assessing the read depth from capture-based NGS data were evaluated. The validation of this workflow has been published recently and highlighted very high sensitivity and positive predictive value for NGS gene panels [27].

### 3.4. Clinical Utility Assessment 

To illustrate the clinical utility of this panel, we report 5 cases in which the NGS approach proved to be significantly more efficient than traditional Sanger sequencing. All the variants identified through the NGS workflow have been confirmed using Sanger sequencing (SNVs and indels) or quantitative multiplex PCR of short fluorescent fragments-QMPSF (CNVs).

Case 1: The LysoGene panel enabled the characterization of 2 pathogenic heterozygous variants in *NPC2* gene. The variant NM_006432.3:c.58G>T-p.(Glu20 *) has been reported in HGMD and has been published [28]. The second frameshift variant, c.87del-p.(Val30Trpfs*5) is novel. The presence of these variants was consistent with the diagnosis of Niemann Pick C type 2 disease. Sanger sequencing of *NPC2* in the parents confirmed allelic segregation.

Case 2: The analysis of the neuronal lipofuscinosis ceroid sub-panel allowed the characterization of two pathogenic heterozygous variants in the *TPP1* gene in this patient. Both variants, NM_000391.3:c.196C>T-p.(Gln66 *) and c.622C>T; p.(Arg208 *), have been reported in HGMD and previously published [29,30]. Allelic segregation was confirmed by the study of the parents’ DNA.

Case 3: Given the clinical picture, priority was given to the analysis of genes involved in pathologies with macular cherry-red spots (Figure 1). Two pathogenic variants were identified in *HEXB*, NM_000521.3:c.1165dup-p.(Gln389Profs*22) which has never been described before, and c.1417+5G>A-p.? predicted to abolish the splicing donor site [31]. Enzymatic activities of hexosaminidase A and total hexosaminidases were greatly reduced in leukocytes and plasma. All these results pointed to Sandhoff disease.

Case 4: NGS sequencing of *HGSNAT* gene succeeded in retrieving the variants inherited from the mother (NM_152419.2:c.234+1G>A-p.? and c.710C>A-p.(Pro237Gln)) and enabled the identification of a heterozygous deletion of exon 15 (NM_152419.2:c.(1464+1_1465-1)_(1542+1_1543-1)del-p.?) which is carried by the paternal allele. This finding made it possible to confirm on a molecular basis the diagnosis of Sanfilippo type C in this patient. 

Case 5: Rapid *GAA* gene sequencing using the LysoGene panel enabled the characterization of two pathogenic heterozygous variants: NM_000152.2:c.-32-13T>G-p.? in intron 1 which has previously been reported in adult form of Pompe disease [32], and c.2238G>C-p.(Trp746Cys) in exon 16 [33]. Sanger sequencing of the parents’ DNA confirmed allelic segregation. Metabolic work up showed a reduced acid maltase activity.

## 4. Discussion

Diagnostic difficulties in LDs arise from the wide clinical, biochemical and molecular heterogeneity observed in these pathologies and highlight the crucial need of multidisciplinary collaboration for the diagnosis and management of these diseases [34,35]. LDs, like other IEMs, are primarily due to monogenic alteration, but a large number of genetic and environmental factors modulate their phenotypic expression and underlie the wide range of clinical severity associated with LDs. This concept has been extended to connect IEMs to common diseases as part of a metabolic disease spectrum. All these pathologies imply necessarily several genes and represent a continuum. Indeed, in IEMs, the influence of one gene is dominant and in common diseases an equivalent contribution of several gene alterations might be observed [36]. In addition, some LDs display phenotypic overlaps that often lead to misdiagnosis. Testing several hypotheses sequentially may result in a delay or failure to succeed in reaching the diagnosis. Of note, some lysosomal hydrolases may have reduced in vitro activity in clinically healthy individuals, referred to as pseudodeficiency. A set of variants known to cause pseudodeficiency has been characterized in the sequences of the corresponding genes that leads to an in vitro instability of the enzyme while the enzyme remains functionally active in vivo [37].

To smooth out and speed up LD screening and diagnosis, a paradigm shift is urgently needed to move from hypothesis-driven to data-driven strategies. Omics approaches along with bioinformatics tools offer a great opportunity to establish a validated workflow enabling the assessment of a large panel of diseases. Subsequently, targeted approach technologies may be used to confirm the identified abnormalities. 

Here, we describe the analytical validation of an NGS-based sequencing panel encompassing 51 genes implicated in LDs. The assay demonstrated a high sensitivity and reliability and was efficient in characterizing both variants involving a small number of nucleotides (SNVs/indels) and large-scale rearrangements (CNVs). By multiplexing patient samples and several genes on a single platform, the limitations related to Sanger sequencing were addressed. This approach allowed for both lowering the costs and enhancing the diagnostic effectiveness. Recent studies reported NGS-based analyses in LD genetic diagnosis [8,9,10,11]. CNV detection was reported in only one study that included 28 LD genes [11]. Of note, the present work enabled the analysis of CNVs, not reachable by Sanger sequencing, for all the included 51 LD-related genes. This markedly broadens the scope of this panel for LD genetic investigations.

To illustrate the clinical integration of our panel, we reported 5 LD patients for which NGS analysis provided with fast and accurate results. 

The NGS panel allowed us to guide the diagnosis toward of Niemann-Pick type C in Case 1, Sandhoff disease in Case 3 and Pompe disease in Case 5 while the clinical pictures were unspecific. In Case 2, the clinical presentation was suggestive of a ceroid lipofuscinosis. A fast molecular diagnosis was critical as a clinical trial for TPP1 deficiency based on intraventricular enzyme replacement therapy was ongoing. To be efficient, this treatment had to be implemented before psychomotor regression [38]. NGS analysis helped in identifying pathogenic variants in *TPP1* gene and the patient was successfully included in the ongoing clinical trial. The clinical utility of simultaneous CNV characterization is exemplified in Case 4. Indeed, the NGS workflow allowed the retrieval of the SNVs located on the maternal allele as well as the characterization of a CNV inherited from the father. Thus, NGS approach enabled the confirmation of this diagnosis on a molecular basis.

## 5. Conclusions

Clinical heterogeneity, phenotypic overlap, and variable age at onset are still major hurdles for fast and effective diagnosis of LDs. Combining NGS-based technology capabilities with efficient bioinformatics workflows offer a promising opportunity to enhance LD characterization through high throughput molecular profiling. Two main driving diagnosis situations stand out: (i) in typical clinical presentation, targeted biochemical profiling is the gold standard informative way to go with a subsequent molecular confirmation; (ii) in challenging clinical situation, first-tier NGS-based molecular profiling seems to be more informative to parse the clinical puzzle. In addition, conventional biochemical profiling confirmation is strongly recommended whenever possible.

## Figures and Tables

**Table 1:** Included genes in the LysoGene panel.

Disease	Inheritance	Gene	NM_
α-glucosidase deficiency	AR	*GAA*	NM_000152.3
α-mannosidase deficiency	AR	*MAN2B1*	NM_000528.3
Aspartylglucosaminidase deficiency	AR	*AGA*	NM_000027.3
β-mannosidase deficiency	AR	*MANBA*	NM_005908.3
α-fucosidase deficiency	AR	*FUCA1*	NM_000147.4
Cathepsin A deficiency	AR	*CTSA*	NM_000308.2
α-*N*-acetylgalactosaminidase deficiency	AR	*NAGA*	NM_000262.2
α-neuraminidase deficiency	AR	*NEU1*	NM_000434.3
Cystinosin deficiency	AR	*CTNS*	NM_004937.2
Lysosome-associated membrane protein 2 deficiency	XL	*LAMP2*	NM_002294.2
Niemann-Pick disease type C1	AR	*NPC1*	NM_000271.4
Niemann-Pick disease type C2	AR	*NPC2*	NM_006432.3
Sialin deficiency	AR	*SLC17A5*	NM_012434.4
Mucolipin 1 deficiency	AR	*MCOLN1*	NM_020533.2
Lysosomal acid lipase deficiency	AR	*LIPA*	NM_000235.2
Cathepsin K deficiency	AR	*CTSK*	NM_000396.3
UDP-*N*-acetylglucosamine-2-epimerase/*N*-acetylmannosamine kinase deficiency	AR	*GNE*	NM_005476.5
UDP-*N*-acetylglucosamine-1-phosphotransferase α/β subunit deficiency	AR	*GNPTAB*	NM_024312.4
α-iduronidase deficiency	AR	*IDUA*	NM_000203.3
Iduronate sulfatase deficiency	XLR	*IDS*	NM_000202.5
Heparan *N*-sulfatase deficiency	AR	*SGSH*	NM_000199.3
*N*-acetylglucosaminidase deficiency	AR	*NAGLU*	NM_000263.3
Heparan-α-glucosaminide *N*-acetyltransferase deficiency	AR	*HGSNAT*	NM_152419.2
*N*-acetylglucosamine 6-sulfatase deficiency	AR	*GNS*	NM_002076.3
*N*-acetylgalactosamine 6-sulfatase deficiency	AR	*GALNS*	NM_000512.4
Hyaluronidase deficiency	AR	*HYAL1*	NM_153281.1
*N*-acetylgalactosamine 4-sulfatase deficiency	AR	*ARSB*	NM_000046.3
β-glucuronidase deficiency	AR	*GUSB*	NM_000181.3
Palmitoyl-protein thioesterase 1 deficiency	AR	*PPT1*	NM_000310.3
Cathepsin D deficiency	AR	*CTSD*	NM_001909.4
Progranulin deficiency	AD, AR	*GRN*	NM_002087.2
Tripeptidyl-peptidase 1 deficiency	AR	*TPP1*	NM_000391.3
CLN3 disease	AR	*CLN3*	NM_001042432.1
CLN4 disease	AD	*DNAJC5*	NM_025219.2
CLN5 disease	AR	*CLN5*	NM_006493.2
CLN6 disease	AR	*CLN6*	NM_017882.2
CLN7 disease	AR	*MFSD8*	NM_152778.2
CLN8 disease	AR	*CLN8*	NM_018941.3
Osteopetrosis	AR	*OSTM1*	NM_014028.3
Formyl-glycine generating enzyme deficiency	AR	*SUMF1*	NM_182760.3
GM2 activator protein deficiency	AR	*GM2A*	NM_000405.4
Arylsulfatase A deficiency	AR	*ARSA*	NM_000487.5
Acid ceramidase deficiency, inflammatory phenotype	AR	*ASAH1*	NM_177924.3
α-Galactosidase A deficiency	XL	*GLA*	NM_000169,2
Glucocerebrosidase deficiency	AR	*GBA*	NM_001005741.2
β-galactosylceramidase deficiency	AR	*GALC*	NM_000153.3
Acid sphingomyelinase deficiency	AR	*SMPD1*	NM_000543.4
β-hexosaminidase β-subunit deficiency	AR	*HEXB*	NM_000521.3
β-hexosaminidase α-subunit deficiency	AR	*HEXA*	NM_000520.4
β-galactosidase deficiency, GM1 gangliosidosis phenotype	AR	*GLB1*	NM_000404.2
Atypical Gaucher disease due to saposin C deficiency	AR	*PSAP*	NM_002778.2

## Data Availability

All the data that support the findings are presented in the manuscript and the Supplementary Materials.

## Supplementary Materials

The following are available online at https://www.mdpi.com/2075-4418/11/2/294/s1, Table S1: cohort molecular data, Table S2: variant effect, Table S3: panel gene classification, Table S4: intra-assay variation assessment, Table S5: inter-assay variation assessment.
